# Regulation of aldosterone secretion by Ca_v_1.3

**DOI:** 10.1038/srep24697

**Published:** 2016-04-21

**Authors:** Catherine B. Xie, Lalarukh Haris Shaikh, Sumedha Garg, Gizem Tanriver, Ada E. D. Teo, Junhua Zhou, Carmela Maniero, Wanfeng Zhao, Soosung Kang, Richard B. Silverman, Elena A. B. Azizan, Morris J. Brown

**Affiliations:** 1 Clinical Pharmacology Unit, University of Cambridge, Box 110, Addenbrooke’s Hospital, Cambridge, CB2 2QQ, UK; 2 Yale School of Medicine, 367 Cedar Street, New Haven, Connecticut 06510, USA; 3 Barts Heart Centre, William Harvey Research Institute, Queen Mary University London, London EC1M 6BQ, UK; 4 Human Research Tissue Bank, Cambridge University Hospitals NHS Foundation Trust, Addenbrooke’s Hospital, Cambridge, CB2 0QQ, UK; 5 Department of Chemistry, Chemistry of Life Processes Institute, and Center for Molecular Innovation and Drug Discovery, Northwestern University, Evanston, Illinois 60208-3113, USA; 6 Department of Medicine, Faculty of Medicine, The National University of Malaysia (UKM) Medical Centre, Kuala Lumpur 56000, Malaysia

## Abstract

Aldosterone-producing adenomas (APAs) vary in phenotype and genotype. Zona glomerulosa (ZG)-like APAs frequently have mutations of an L-type calcium channel (LTCC) Ca_V_1.3. Using a novel antagonist of Ca_V_1.3, compound **8**, we investigated the role of Ca_V_1.3 on steroidogenesis in the human adrenocortical cell line, H295R, and in primary human adrenal cells. This investigational drug was compared with the common antihypertensive drug nifedipine, which has 4.5-fold selectivity for the vascular LTCC, Ca_V_1.2, over Ca_V_1.3. In H295R cells transfected with wild-type or mutant Ca_V_1.3 channels, the latter produced more aldosterone than wild-type, which was ameliorated by 100 μM of compound **8**. In primary adrenal and non-transfected H295R cells, compound **8** decreased aldosterone production similar to high concentration of nifedipine (100 μM). Selective Ca_V_1.3 blockade may offer a novel way of treating primary hyperaldosteronism, which avoids the vascular side effects of Ca_V_1.2-blockade, and provides targeted treatment for ZG-like APAs with mutations of Ca_V_1.3.

Aldosterone-producing adenomas (APAs), which arise from the adrenal cortex, are one of the most common curable causes of hypertension^1^,^2^,^3^. They account for approximately half of primary aldosteronism, which is estimated to be present in 5–13% of all hypertensive patients, and in at least 20% of patients with resistant hypertension^4^. However, it is likely that fewer than 10% of APAs are ever diagnosed; and fewer still are removed in time to cure hypertension and prevent resistance to effective drug treatment^2^,^5^.

We previously reported somatic gain-of-function mutations in two genes that regulate Na^+^, K^+^ and Ca^2+^ transport in APAs with a zona glomerulosa (ZG)-like phenotype^6^. Whole exome sequencing of small-cell APAs with a ZG-like gene expression profile found five out of ten to harbour one of four different somatic mutations in the voltage dependent L-type Ca^2+^ channel, Ca_V_1.3 (encoded by the gene *CACNA1D*). These four substitution mutations, V259D, G403R, I750M, and P1336R, cluster around the Ca^2+^ pore between the S5 and S6 domains that line the inner pore surface. The mutations occur in conserved sites within functional domains such as the voltage-sensing domain to the pore (V259D and P1336R) and the channel activation gate (G403R and I750M)^6^. The G403R and I750M mutations were simultaneously reported as rare de novo germline mutations presenting at birth, together with several patients having somatic mutations of the same residues in sporadic APAs^7^. Our own replication sequencing revealed three further mutations, and sequencing of APAs in a large European consortium has now identified a total of 19 somatic mutations in or near one of the four Ca^2+^ channel pore-forming domains 6,8. Patch clamping of HEK293 cells has shown that at least 6 of the 19 mutations affect the Ca_V_1.3 channel function and allow for increased Ca^2+^ influx through either shifting voltage-dependent activation towards more negative voltages, decelerating inactivation, and/or increasing currents through a higher open channel probability^6^,^9^.

The current medical treatment of primary hyperaldosteronism is blockade of the mineralocorticoid receptor, which can lead to an increase in aldosterone secretion^10^. Therefore, blockade of calcium entry through selective antagonism of Ca_V_1.3 might present a valuable therapeutic target. We therefore aimed to investigate whether Ca_V_1.3 mutations cause the postulated increase in aldosterone secretion from human adrenocortical cells, and whether blockade of calcium entry reverses this. We studied the potential value of this target using 1-(3-chlorophenethyl)-3-cyclopentylpyrimidine-2,4,6-(1H,3H,5H)-trione (compound **8**), which was found to be more than 600 times more selective for Ca_V_1.3 than Ca_V_1.2^11^. Nifedipine, a common antihypertensive drug, was used in comparison as a non-selective, or slightly Ca_V_1.2 selective antagonist of L-type calcium channels (IC_50_ = 0.016 μM)^12^,^13^,^14^. We also undertook immunohistochemistry of normal human adrenals, and APAs, in order to determine whether Ca_V_1.3 is a ZG-selective L-type Ca^2+^ channel and whether blockade may have greater expected effect on aldosterone secretion from APAs than from normal adrenal.

To study the role of Ca_V_1.3 on aldosterone secretion, we first investigated the substitution mutations near the voltage-sensing domain, P1336R and V259D, on 24-h aldosterone production in transiently transfected H295R cells to find if the different changes seen in our electrophysiology data translated to changes in aldosterone secretion^6^. We then contrasted the aldosterone secretion of cells transfected with mutant Ca_V_1.3 channels to those transfected with wild-type Ca_V_1.3 channel in the presence of compound **8** or nifedipine to study if blockade of calcium entry affects APAs with a Ca_V_1.3 mutation differently. Transfection of H295R cells with exogenous Ca_V_1.3 was performed with β_3_ and α_2_δ accessory subunits, the subunits we used previously to show gain-of function effects of the mutations on Ca^2+^ currents^6^. As transfected channels and subunits do not necessarily emulate *in vivo* expression, we also tested the effect of compound **8** and nifedipine on endogenous Ca_V_1.3 present in H295R cells and primary adrenal cells acquired from adrenals containing an APA (both tumour and adjacent normal adrenal tissues).

## Results

### Ca_V_1.3 mutations and compound 8 alter aldosterone production

**Figure 1:**
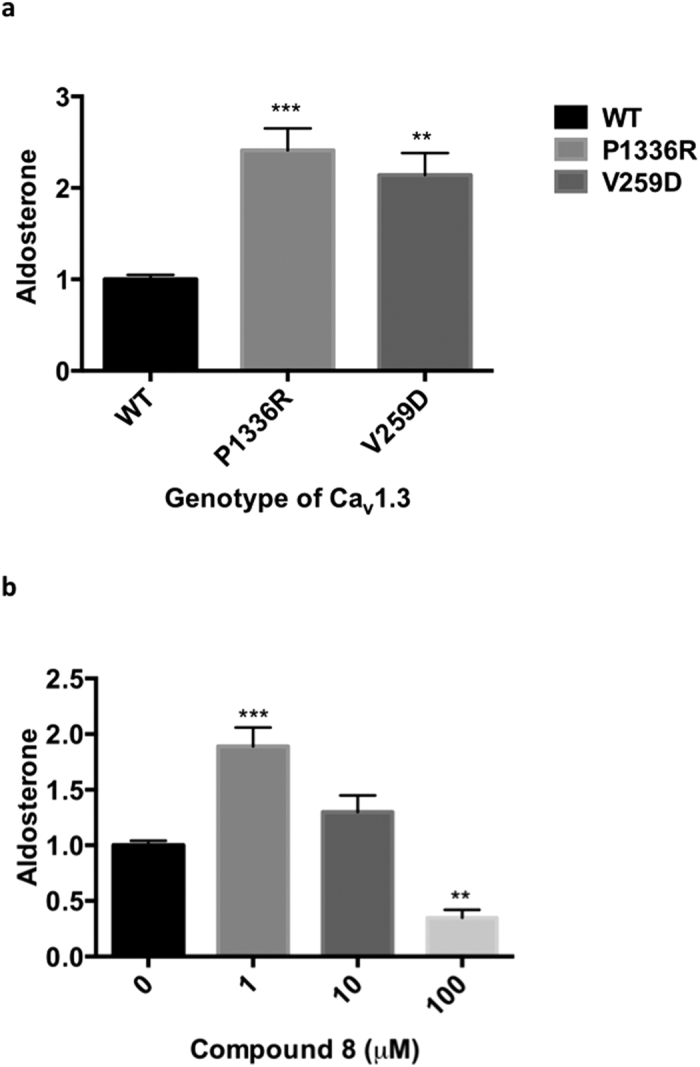
Ca_V_1.3 mutations and compound 8 alter aldosterone production. Comparison of stimulated aldosterone production in (**a**) wild-type (WT), P1336R, and V259D Ca_V_1.3 transfected H295R cells (n = 5) and in (**b**) different concentration of compound 8 on WT H295R cells (n = 3). Student t-test was used to calculate significance. **P < 0.01 and ***P < 0.001, compared to baseline (Wild-type or 0 M compound 8). The *n* value represents number of separate experiment/transfection performed. Each experiment/transfection had 6 biological replicates. Aldosterone results shown here were measured by RIA method and are relative to basal level (Wild-type or 0 M compound 8).

Transfection of H295R cells with Ca_V_1.3 mutants P1336R and V259D caused a 2.4 ± 0.2 (*P* = 0.0004) and 2.1 ± 0.2 (*P* = 0.002) fold increase, respectively, in basal aldosterone production compared to wild-type transfected H295R cells (Fig. 1a) and similarly in angiotensin II stimulated aldosterone production (Supplementary Fig. 1).

Exposure of H295R cells transfected with wild-type Ca_V_1.3 to low concentration (1 μM) of compound **8** almost doubled aldosterone secretion (P = 0.007), whereas high concentration (100 μM) of compound **8** decreased aldosterone production to 35 ± 0.1% of basal level (*P* = 0.003, Fig. 1b).

### Effect of calcium blockade on Ca_V_1.3 genotypes

**Figure 2:**
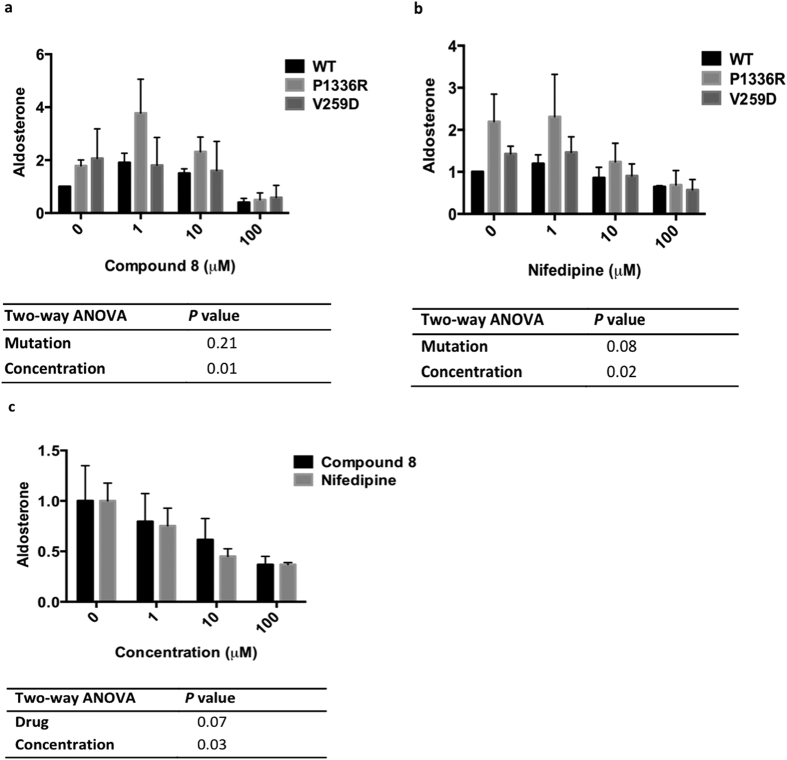
Effect of compound 8 on aldosterone production of different Ca_V_1.3 genotype (**a**) Stimulated aldosterone secretion (n = 3) in the presence of compound 8 and (**b**) stimulated aldosterone secretion in the presence of nifedipine (n = 3) on WT, P1336R and V259D Ca_V_1.3 transfected H295R cells. There was a similar biphasic effect of compound 8 on aldosterone secretion from the mutant P1336R cells (*P* = 0.02; Student’s *t*-test), as that seen in wild-type Ca_V_1.3, but not so in mutant V259D cells or when transfected cells were treated with nifedipine. (**c**) Comparison of basal aldosterone production of non-transfected H295R cells in the presence of 0–100 μM of compound 8 or nifedipine (n = 3). Two-way ANOVA was used to calculate overall significance. Table of *P*-values shows significance of mutation status (Mutation), concentration of treatment (Concentration), and type of treatment (Drug), on aldosterone production. The *n* value represents number of separate experiment/transfection performed. Each experiment/transfection had 6 biological replicates. Aldosterone was measured by RIA (**a**,**b**) or RIA and HTR-FRET (**c**) method. Results of both methods are relative to basal level (Wild-type or 0 µM of treatment).

In cells transiently transfected with wild-type, P1336R, or V259D Ca_V_1.3, there was a similar biphasic effect of compound **8** on aldosterone secretion from the mutant P1336R cells, as that seen in wild-type Ca_V_1.3. In mutant V259D cells, compound 8 was inhibitory only at 100 μM (Fig. 2a). Using the same protocol as for compound **8**, the inhibitory effect of nifedipine on aldosterone secretion from Ca_V_1.3 transfected H295R cells was determined. In wild-type Ca_V_1.3 transfected cells, after treatment with 1 μM, 10 μM or 100 μM nifedipine, a 35 ± 12% decrease of aldosterone secretion was observed only at the highest concentration of nifedipine interrogated (100 μM)(*P* = 0.0001, Fig. 2b) a considerable excess of its K_i_ for Ca_V_1.2 (IC_50_ = 0.016 μM)^12^,^13^. In P1336R and V259D transfected cells, despite the increased aldosterone secretion compared to that of wild-type cells (as seen at 0 μM), the presence of high concentration of nifedipine (100 μM) decreased aldosterone production similarly across all genotypes (Fig. 2b).

In non-transfected H295R cells (with only endogenous Ca_V_1.3 and endogenous Ca_V_1.3 accessory subunits present), compound 8 and nifedipine, 1–100 μM, decreased basal aldosterone secretion (Fig. 2c).

### Compound 8 decreases aldosterone production in primary human adrenal cells

**Figure 3:**
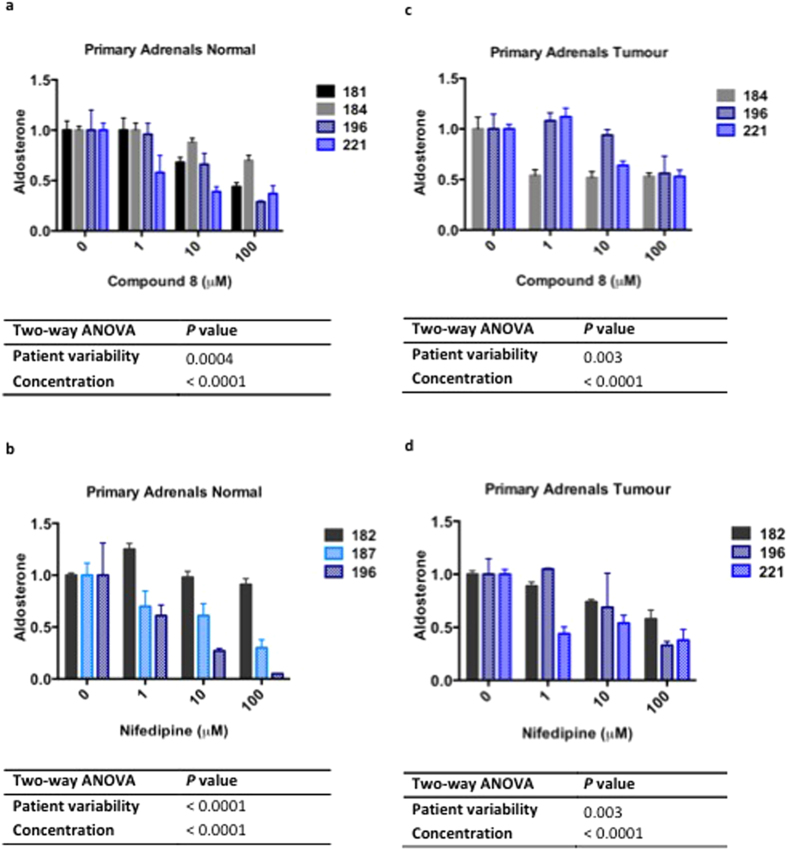
Compound 8 decreases aldosterone production in primary human adrenal cells. Aldosterone secretion of (**a,b**) normal primary adrenal cells or (**c,d**) aldosterone-producing adenomas (APAs) in the presence of compound 8 (**a,c**) or nifedipine (**b,d**). Dose response curve between 0-100 μM of compound **8** on (**a**) normal primary adrenal cells (n = 4) and (**c**) APA cells (n = 3), and nifedipine on (**b**) normal primary adrenal cells (n = 3) and (**d**) APA cells (n = 3). Two-way ANOVA was used to calculate overall significance. Table of *P*-values shows significance of patient differences (Patient variability) and concentration of treatment (Concentration) on aldosterone production. The *n* value represents number of individual patient samples used for each experiment. Each concentration was replicated 2–12 times within each individual patient samples (which depended on quantity of primary cells available). Aldosterone results shown here are relative to 0 M of treatment. Numbers 181, 182, 184, 187, 196, and 221 represent individual patient ID. Clinical data for these patients is provided in Supplementary Table 1. Primary cell cultures from patients 181, 182, and 184 were performed in the absence of angiotensin II (seen as solid bars) whereas primary cell cultures 187, 196, and 221 were stimulated with 10 nM angiotensin II (seen as hatched bars). Aldosterone was measured by RIA and ELISA method.

In primary human adrenal cells cultured from the normal adjacent adrenals of patients with an APA, 10 and 100 μM of compound **8** inhibited aldosterone production by 35 ± 10 and 43 ± 11%, respectively (*P* < 0.05; Fig. 3a). Cortisol secretion was also decreased to 72 ± 1 and 50 ± 4% of basal level, respectively (*P* < 0.05; Supplementary Fig. 2). As for nifedipine, effect on aldosterone production was varied - not all cell cultures from the different patients showed a reduction, even at the high concentration of 100 μM (Fig. 3b).

In APA cells, with increasing concentrations of 1, 10 and 100 μM, both compound **8** and nifedipine showed a dose dependent decrease in aldosterone production, to a minimum average of 54 ± 2 and 43 ± 13% of basal level, respectively (*P* < 0.005; Fig. 3c,d).

### Localization of Ca_V_1.3 in adrenals containing an APA

**Figure 4:**
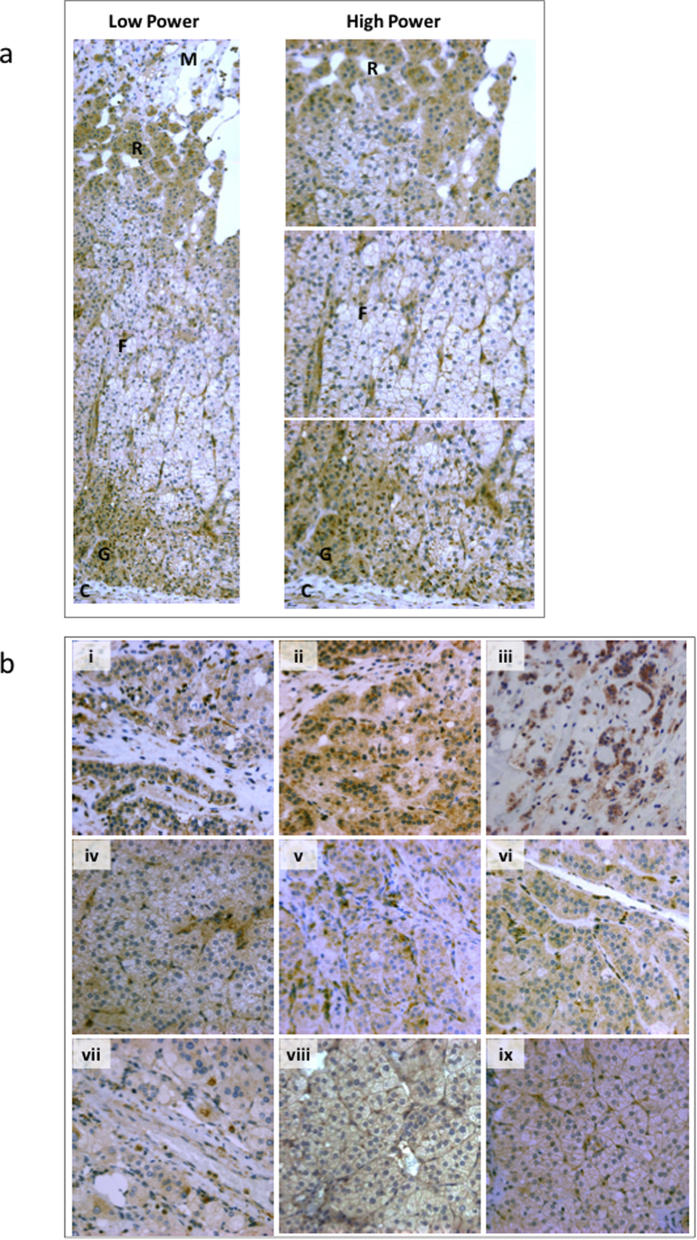
Localization of Ca_V_1.3 in human adrenals. (**a**) Immunohistochemistry (IHC) of Ca_V_1.3 on formalin-fixed paraffin-embedded (FFPE) adrenal sections localized the channel to the zona glomerulosa (ZG) and zona reticularis (ZR) of the adrenals. In the ZG, cytoplasmic and juxtanuclear accumulation of Ca_V_1.3 was observed whereas in the ZR, staining was mainly cytoplasmic. Picture is representative of 12 normal adjacent adrenal glands, 3 from patients with a phaeochromocytoma and 9 from patients with an aldosterone-producing adenoma (APA). C, capsule; G, zona glomerulosa; F, zona fasciculata; R, zona reticularis; M, adrenal medulla. (**b**) Ca_V_1.3 expression in APA cells. IHC of Ca_V_1.3 on FFPE adrenal sections were performed on three different types of APAs: (i–iii) ZG-like (low nucleus to cytoplasm ratio) APAs without a Ca_V_1.3 mutation, (iv–vi) APAs with a Ca_V_1.3 mutation, and (vii–ix) APAs with a *KCNJ5* mutation. Immunostaining reveals a mixture of cytoplasm and membranous sublocalization in APA cells.

In sections of adjacent normal adrenal, that were adjacent to an APA or pheochromocytoma, Ca_V_1.3 was detected in the ZG and the zona reticularis (ZR) (Fig. 4a). Only in ZG were juxtanuclear accumulation seen (as shown in the zoomed image), as ZR staining was mainly cytoplasmic (Fig. 4a). Exogenous Ca_V_1.3 in H295R transfected cells had mainly membranous expression (Supplementary Fig. 3).

In APAs, different patterns of Ca_V_1.3 expression were observed. Ca_V_1.3 was expressed at the cell membrane, cytoplasmic, at the edge of cell clusters, or sparsely, or not at all (Fig. 4b and Supplementary Fig. 4).

## Discussion

We previously reported that somatic mutation of Ca_V_1.3 is present in a subset of APAs, distinguished by several features resembling normal ZG^6^. In a large multi-centre study of 474 APAs, the frequency of Ca_V_1.3 mutation was estimated to be 9.3%^8^. Although no particular histological phenotype was found in the multi-centre study^8^, one centre within the study did subsequently report that of their 71 APAs, Ca_V_1.3 mutant APAs (3 of 71) were composed mainly of ZG-like cells^15^. Thus the current approximation could be a substantial underestimation since (a) half of our selected ZG-like APAs that were exome sequenced had a Ca_V_1.3 mutation^6^; and (b) our experience is that such tumours are frequently too small to be detected by conventional adrenal imaging. We therefore wished to show whether the mutations are likely to increase aldosterone production, rather than trigger development of the adenoma, and whether this increase could be reversed by blockade of calcium entry. As few APAs are diagnosed in time to offer high likelihood of surgical cure from hypertension, and the increased recognition of aldosterone morbidity, a need arises for novel therapies that suppress aldosterone production, and lack the adverse effects of aldosterone receptor blockade and other less specific therapies for hypertension.

Herein we report that the two Ca_V_1.3 mutations studied, selected for having different electrophysiological effects^6^, do increase aldosterone secretion of transfected human adrenocortical cells (Fig. 1). Furthermore, calcium blockade using compound **8,** an investigational Ca_V_1.3 inhibitor, and nifedipine, a non-selective L-type calcium channel inhibitor, reversed the increase (Fig. 2). The inhibition of aldosterone secretion was seen in the presence of the highest concentration of compound **8** interrogated in this study (100 μM) in H295R cells transfected with Ca_V_1.3 mutants (Fig. 2); whereas in non-transfected H295R cells and primary adrenal cells, inhibition of aldosterone secretion could be seen at lower concentrations (1 and 10 μM) (Figs 2c and 3d). We also postulate that regardless of whether a given APA has a somatic mutation of Ca_V_1.3, the channel is often more active than in normal ZG cells, where immunohistochemistry suggests Ca_V_1.3 is mainly internalised (Fig. 4).

Compound **8** was interrogated in this study as it was found to be the most selective Ca_V_1.3 antagonist among 60,480 commercial compounds and a few hundred non-commercial compounds (Silverman lab) tested for efficacy in blocking Ca_V_1.3 or Ca_V_1.2 in stably transfected HEK293 cells. Compound **8** was reported to inhibit Ca_V_1.3 > 600-fold more potently than Ca_V_1.2^11^. Subsequent studies have questioned this degree of selectivity, and even whether compound **8** is an agonist or antagonist^16^,^17^. Nevertheless, it is well known that the effects of L-type Ca^2+^ channel blockade can differ among tissues depending on factors such as resting membrane potential^18^. Consequently, the hyperpolarisation of adrenocortical cells may have enhanced our ability to detect an antagonist effect of compound **8**. Further, we may have fortuitously selected the *CACNB* isoform which maximises compound **8** selectivity, namely *CACNB3* (encoding for the β_3_ subunit). In subsequent analysis, however, we found *CACNB2* to be the predominant isoform in human adrenal, indeed being one of the genes most up-regulated in ZG compared to zona fasciculata (ZF)^19^. Thus, for the pharmacological responses of the different mutations to be legitimately compared, a better Ca_V_1.3 antagonist than Compound **8** is needed. Future antagonists should be developed not only based on its selectivity for Ca_V_1.3 but also on its functionality with the prevalent accessory subunits in the human adrenal.

In our cells transfected with exogenous Ca_V_1.3, the stimulatory effect of apparent low dose calcium blockade on aldosterone secretion was observed only for Compound **8**, but not nifedipine. This increase in aldosterone secretion could have been due to low dose compound **8** behaving as a channel activator^16^; but toxicity (and hence leakage of aldosterone) due to high calcium influx in transfected H295R cells cannot be dismissed, since no stimulation of aldosterone secretion was seen in untransfected cells (Fig. 2c). The limitation of our expression Ca_V_1.3 model, however, was that the cell line we used, H295R cells, express a mixture of endogenous Ca_V_1.2 and Ca_V_1.3 whereas primary human ZG cells express mainly Ca_V_1.3^19^,^20^. Moreover, the immortalised H295R cells were not a perfect model for primary aldosteronism as other adrenal corticosteroids are secreted^21^. This cell line was used mainly due to the ease of transfecting exogenous mutant Ca_V_1.3. Hence, to supplement our transfection experiments, not only was compound 8 also studied in un-transfected H295R cells, but also in primary adrenal cells (of which we have a limited supply), to support endogenous Ca_V_1.3 role in aldosterone regulation. To note, as we did not find a linear relationship between increase in aldosterone production and amount of transfected constructs, no correction for transfection efficiency whether by Western blots or qPCR was performed. Transfection rates of exogenous Ca_V_1.3 were confirmed as similar visually, using its GFP-tag.

Previous studies have shown a number of dihydropyridines to reduce aldosterone secretion from adrenocortical cells^22^. We chose nifedipine as a comparator because of experience with its use in patients, in whom it was the first dihydropyridine to be used^23^,^24^,^25^, and also because of its modest Ca_V_1.2 selectivity. Nifedipine is expected to exert its Ca_V_1.2 blockade at concentrations around 4.45 nM^14^. At the lowest concentration of nifedipine that we had interrogated (1 μM), a concentration which should have easily blocked Ca_V_1.2, only some inhibition of aldosterone could be seen in non-transfected H295R and primary adrenal cells and none at all in H295R cells transfected with Ca_V_1.3 mutants (Figs 2 and 3). The shallow concentration-response curves are consistent with blockade of different sites at low and high concentrations (Figs 2 and 3). Dihydropyridines sometimes cause substantial reductions in plasma aldosterone in patients with primary aldosteronism^26^. However this is not the predominant response at usual clinical doses, and increasing the dose to the presumed Ca_V_1.3-blocking range is precluded by the vascular side effects, particularly peripheral edema^25^,^27^.

The potential attraction of selective Ca_V_1.3 blockade is that such a drug can be used at a dose which achieves substantial suppression of aldosterone secretion, without the vascular side effects of currently used L-type Ca^2+^ blockers^25^,^27^. Previously, a T-type Ca^2+^ channel blocker, mibefradil, was introduced whose reduction in aldosterone secretion was among the theoretical advantages over L-type Ca^2+^ blockade^28^; however the drug was withdrawn due to reports of dangerous and even fatal interactions with other drugs and was later found to cause serious effects on QTc^29^. *In vitro* studies, have shown that single blockade of either L-type or T-type Ca^2+^ channels can decrease aldosterone production, even though the influx of Ca^2+^ in the ZG is thought to be mediated by both channels^28^,^30^,^31^,^32^. While there has also been considerable attempt to develop inhibitors of aldosterone synthase as a therapeutic class^33^, these have foundered on the challenge of developing a drug, which inhibits aldosterone synthase, without effect on the 95% homologous enzyme catalysing cortisol synthesis (encoded by the gene *CYP11B1*). By contrast, the homology between Ca_V_1.2 and Ca_V_1.3 is only 75%^34^. Thus, even though compound **8** itself may not be the ideal drug candidate to progress for treatment of hyperaldosteronism, there are a number of sites outside the dihydropyridine-binding site where Ca_V_1.2 and Ca_V_1.3 differ sufficiently to suggest that selective blockade is achievable.

Three drugs do have clinical efficacy in patients with primary aldosteronism: spironolactone, eplerenone and amiloride^35^,^36^. However, the efficacy of the latter two is modest, and the use of spironolactone is limited in men by the anti-androgenic effects of higher doses^37^,^38^. All three drugs cause substantial increases in plasma aldosterone secretion, probably secondary to the rise in plasma K^+^, and there is some concern whether aldosterone could have adverse vascular effects through a non-canonical aldosterone receptor^39^,^40^. Although no evidence exists in humans, there is an additional theoretical benefit from blocking aldosterone synthesis rather than response – that such a drug could cause involution of aldosterone-producing cells. This is suggested by the observation that genetic deletion of the enzyme aldosterone synthase leads to apoptosis of the normal ZG cells^41^.

In summary, we previously reported ZG-like APAs to have Ca_V_1.3 mutations. In this study, we confirmed that Ca_V_1.3 is localized to the human adrenal ZG. By blocking endogenous Ca_V_1.3 in primary human adrenal and transfecting mutant Ca_V_1.3 in the human adrenocortical cell line, H295R, we have also confirmed that Ca_V_1.3 plays a role in human adrenal steroidogenesis. Taken together, these discoveries suggest that Ca_V_1.3 can provide a novel mechanism and target for regulating excess aldosterone secretion and may be a novel way of treating hyperaldosteronism, especially those caused by ZG-like APAs with a Ca_V_1.3 mutation. Since non-selective or Ca_V_1.2 selective dihydropyridines are dose-limited clinically by vascular effects, a selective Ca_V_1.3 antagonist may be valuable for suppressing aldosterone secretion in some patients with aldosterone-dependent hypertension.

## Methods

### Cell culture experimentation

H295R cells, were cultured in growth medium consisting of DMEM/Nutrient F-12 Ham supplemented with 10% foetal bovine serum, 100 U of penicillin, 0.1 mg/mL streptomycin, 0.4 mM L-glutamine and insulin-transferrin-sodium selenite medium (ITS) at 37 °C in 5% CO_2_.

For transient transfection, wild-type or mutant P1336R or V259D Ca_V_1.3 GFP-tagged constructs were co-transfected together with constructs for β_3_ and α_2_δ auxiliary subunits of Ca_V_1.3 into H295R cells using Amaxa Nucleofector kit R (Lonza, Germany) with electroporation program P20. The GFP-Ca_V_1.3 wild-type and mutant vectors were obtained from our collaborators; Dr. Jöerg Striessnig’s group at University of Innsbruck Center for Chemistry and Biomedicine, Austria. These constructs were derived from the ‘long’ isoform of the Ca_V_1.3 α_1_ pore-forming subunit, with isoform A of the alternatively spliced exon **8**. Transfected cells were seeded into 24-well plates at 100, 000 cells per well in 0.5 mL of growth medium. At 24-h post-transfection, H295R cells were serum deprived in un-supplemented DMEM/Nutrient F-12 Ham medium for 24-h. At 48-h post-transfection, the transfection efficiency was visualised and qualitatively quantified by fluorescence microscopy. Further experiments were performed on cells with 60–80% transfection efficiency.

For primary cell culture, adrenocortical cells were obtained from the adrenals of patients with Conn’s syndrome that had undergone adrenalectomy at Addenbrooke’s Hospital, Cambridge, UK (Supplementary Table 1). Local ethical approval and informed consent were obtained for each patient and the procedures followed were in accordance with institutional guidelines. After macroscopic identification of APA and adjacent normal adrenal by a trained histopathologist, tissue samples were placed in growth medium within 45 minutes of surgical excision. The APA and adjacent normal adrenal was then digested separately in 3.3 mg/ml collagenase at 37 °C for 2-h. Within a week of procurement, the primary human adrenocortical cells were then randomly seeded into 24-well plates at 50, 000 cells per well in 0.5 mL of growth medium and allowed to settle for a further 48-h before drug treatments were performed.

### Drug treatments with Ca_V_1.3 selective antagonist, compound 8, and L-type calcium blocker, nifedipine

Compound **8** and nifedipine (Sigma-Aldrich, UK) were reconstituted in DMSO to stock concentrations of 1, 10, and 100 mM. Stock concentrations were further diluted (1:1000) in sterile un-supplemented DMEM/Nutrient F-12 Ham for treatments.

Transfected H295R cells were treated at 48-h post transfection (after 24-h of serum deprivation) with either vehicle or compound **8** or nifedipine in un-supplemented DMEM/Nutrient F-12 Ham medium in the presence of 10 nM angiotensin II. Supernatant and cells were harvested after 24-h incubation at 37 °C.

For non-transfected H295R cells, cells were seeded into 24-well plates at 100 000 cells per well in 0.5 mL of growth medium, serum deprived for 24-h, and treated with either vehicle, compound **8** or nifedipine in un-supplemented DMEM/Nutrient F-12 Ham medium. Supernatant and H295R cells were harvested after 24-h incubation at 37 °C.

For primary human adrenal cells, APA and adjacent normal adrenal cells were serum deprived for 24-h, and treated with either vehicle or compound **8** or nifedipine in un-supplemented DMEM/Nutrient F-12 Ham medium in the presence or absence of 10 nM angiotensin II. Supernatant and H295R cells were harvested after 24-h incubation at 37 °C.

### Immunohistochemistry

Immunohistochemistry was performed on formalin-fixed, paraffin-embedded adrenal sections (4 μm) using an automated immunostainer with cover tile technology (Bond-III system, Leica Biosystems). A commercial antibody of Ca_V_1.3, clone N38/8 (UC Davis/NIH NeuroMab Facility; 1:500 dilution), was used as the primary antibody. Negative control experiments, in which the primary antibody was omitted, resulted in a complete absence of staining. Images were captured using a standard bright-field microscope, a U-TV1-X digital camera and CellD software (Olympus UK).

### Confocal Imaging

H295R cells were cultured in complete media on sterilised and poly L-lysine coated cover-slips at the density of 10^5^ cells/well in 12-well cell-culture plate for 24-h. Cells were serum-starved overnight before transfection. Serum-free media was replaced with antibiotic-free serum-containing media at the time of transfection with Lipofectamine 3000 transfection reagent (Life Technologies). Cells were co-transfected with GFP-tagged Ca_V_1.3 WT, β_3_ & α_2_δ constructs according to manufacturer’s instructions. 48-h later plasma membranes of cells were stained with 2ug/ml Wheat Germ Agglutinin, Alexa Fluor^®^ 633 Conjugate (W21404, Life Technologies) in complete media for 10 min at 37 °C. Cells were washed twice with PBS (5 min each), followed by fixing with 4% paraformaldehyde and permeabilisation with 1% trition-X100 (PBST), 10 min each at room temperature. Cells were incubated with blocking buffer (3% BSA in PBS) for 1-h at room temperature and overnight with the Ca_V_1.3 antibody, clone N38/8 (UC Davis/NIH NeuroMab Facility; 1:500 dilution) in 3% BSA-PBST. Goat anti-mouse IgG, Alexa Fluor^®^ 568 Conjugate (A11004, Life Technologies) was used as secondary antibody at 1:1000 dilution in 3%BSA-PBST for 1-h at room temperature. Finally cells were washed thrice in PBST and cover-slips were mounted on slides using VECTASHIELD Antifade Mounting Medium with DAPI (H-1200, Vector Laboratories). Confocal images were taken using Zeiss LSM510 Meta confocal microscope and analysed using Zen 2011 software.

### Aldosterone concentration measurements

Aldosterone concentration was quantitatively measured using three methods due to availability of the kits; Coat-A-Count^®^ Aldosterone (Siemens Medical Solutions, USA), a^125^I solid-phase radioimmunoassay and after the discontinuation of this kit, an ELISA method adapted from researchers in Gomez-Sanchez’s group and finally a commercially available Homogenous Time Resolved Fluorescence Resonance Energy Transfer (HTR-FRET) assay from Cisbio Bioassays, France (used according to manufacturer’s instructions). ELISA was carried out using a selective validated aldosterone monoclonal antibody gifted to us and produced by Gomez-Sanchez’s lab, USA^42^. The aldosterone concentrations from transfected H295R cells were normalized to total cell protein, which was determined by performing the bicinchoninic acid (BCA) protein assay (Pierce Biotechnology, USA).

### Statistical analysis

Experiments were performed with vehicle/plasmid controls where appropriate. Each experiment was performed with biological replicates and the averages were calculated. Aldosterone measurements are expressed as a ratio of basal (control) for each experiment. Results are shown as mean values ± SEM of separate experiments/transfections unless stated otherwise. Statistical analysis, two-tailed Student’s *t*-tests or analysis of variance, was performed as indicated using the standard statistical software, Prism 6 (GraphPad Software, Inc).

## Additional Information

**How to cite this article**: Xie, C. B. *et al*. Regulation of aldosterone secretion by Ca_V_1.3. *Sci. Rep.* **6**, 24697; doi: 10.1038/srep24697 (2016).

## Supplementary Material

Supplementary Information
