# Supplementary material for: Regulation of aldosterone secretion by Cav1.3
Source: Sci Rep. 2016 Apr 21;6:24697. doi: 10.1038/srep24697 (PMC4876952; doi:10.1038/srep24697)
Supplement: Supplementary Information [file srep24697-s1.doc]

**Supplementary figures for:**

**Regulation of aldosterone secretion by Ca_v_1.3**

Catherine B Xie^1†^, Lalarukh Haris Shaikh^1†^, Sumedha Garg^1^, Gizem Tanriver^1^, Ada ED Teo^1^, Junhua Zhou^1^, Carmen Maniero^1^, Wanfeng Zhao^2^, Soosung Kang^3^, Richard B Silverman^3^, Elena AB Azizan^1, 4*^, Morris J Brown^1*^.

^1^Clinical Pharmacology Unit, University of Cambridge, Box 110, Addenbrooke’s Hospital, Cambridge, CB2 2QQ, UK.

^2^Human Research Tissue Bank, Cambridge University Hospitals NHS Foundation Trust, Addenbrooke’s Hospital, Cambridge, CB2 0QQ, UK.

^3^Department of Chemistry, Chemistry of Life Processes Institute, and Center for Molecular Innovation and Drug Discovery, Northwestern University, Evanston, Illinois 60208-3113, USA.

^4^Department of Medicine, Faculty of Medicine, The National University of Malaysia (UKM) Medical Centre, Kuala Lumpur 56000, Malaysia.

^†^,^*^ These authors contributed equally to this work.

**Corresponding author:** Professor Morris J. Brown^1^

Tel: +44 (0)2078 823901; email: morris.brown@qmul.ac.uk

This PDF contains:

Supplementary Reference 43

Supplementary Table 1

Supplementary Fig. 1-4

**Supplementary references**

43 Burton, T. J. *et al.* Evaluation of the sensitivity and specificity of (11)C-metomidate positron emission tomography (PET)-CT for lateralizing aldosterone secretion by Conn's adenomas. *J Clin Endocrinol Metab* **97**, 100-109, doi:10.1210/jc.2011-1537 (2012).

**Supplementary Table 1: Clinical data of patients that provided the adrenals for primary cell culture experimentation**

*Lateralisation index was calculated using SUV ratio (instead of AVS ratio) measured by an ^11^C-metomidate PET-CT scan as previously described[^43^](#_ENREF_1). The SUV ratio >1.25 was found to be equivalent to an AVS ratio >4.

| **Patient ID** | **Age** | **Sex** | **Blood Pressure (mmHg)** | | **Aldosterone (pmol/L)** | | **Renin**  **(IU/mL)** | | **K^+^**  **(mmol/L)** | | **No. of anti-hypertensives** | | **Lateralisation index ratio (adrenal side)** | **Adenoma diameter (mm)** |
| --- | --- | --- | --- | --- | --- | --- | --- | --- | --- | --- | --- | --- | --- | --- |
|  |  |  | ***Pre*** | ***Post*** | ***Pre*** | ***Post*** | ***Pre*** | ***Post*** | ***Pre*** | ***Post*** | ***Pre*** | ***Post*** |  |  |
| **181** | 44 | M | 148/96 | 130/75 | 1043 | 171 | 3 | 20 | 3.5 | 4.7 | 3 | 0 | 50.7 (Right) | 7 |
| **182** | 41 | M | 130/80 | 120/80 | 760 | 65 | 2 | 5 | 3.8 | 4.5 | 2 | 0 | 19.5 (Left) | 10 |
| **184** | 48 | M | 146/103 | 120/70 | 647 | 143 | 5 | 27 | 3.7 | 5 | 3 | 0 | 15 (Right) | 6 |
| **187** | 33 | M | 150/100 | 126/93 | 335 | 192 | 2 | 78 | 3.9 | 4.6 | 3 | 0 | 5.5 (Left) | 10 |
| **196** | 63 | M | 140/90 | 130/85 | 3030 | 96 | 16 | 33 | 3.5 | 4.7 | 3 | 0 | 2.6* (Right) | 11 |
| **221** | 62 | M | 164/86 | - | 336 | - | 2 | - | 3.2 | - | 1 | - | 1.6* (Left) | 18 |

Note: Patient ID 221 is a recent case (post-adrenalectomy visit is due in the future).

*Pre*: pre-adrenalectomy. *Post*: post-adrenalectomy.

AVS: adrenal vein sampling. SUV: Standardized Uptake Values.

**Supplementary Fig. 1: Ca_V_1.3 mutations alter aldosterone production in the absence and presence of angiotensin II**


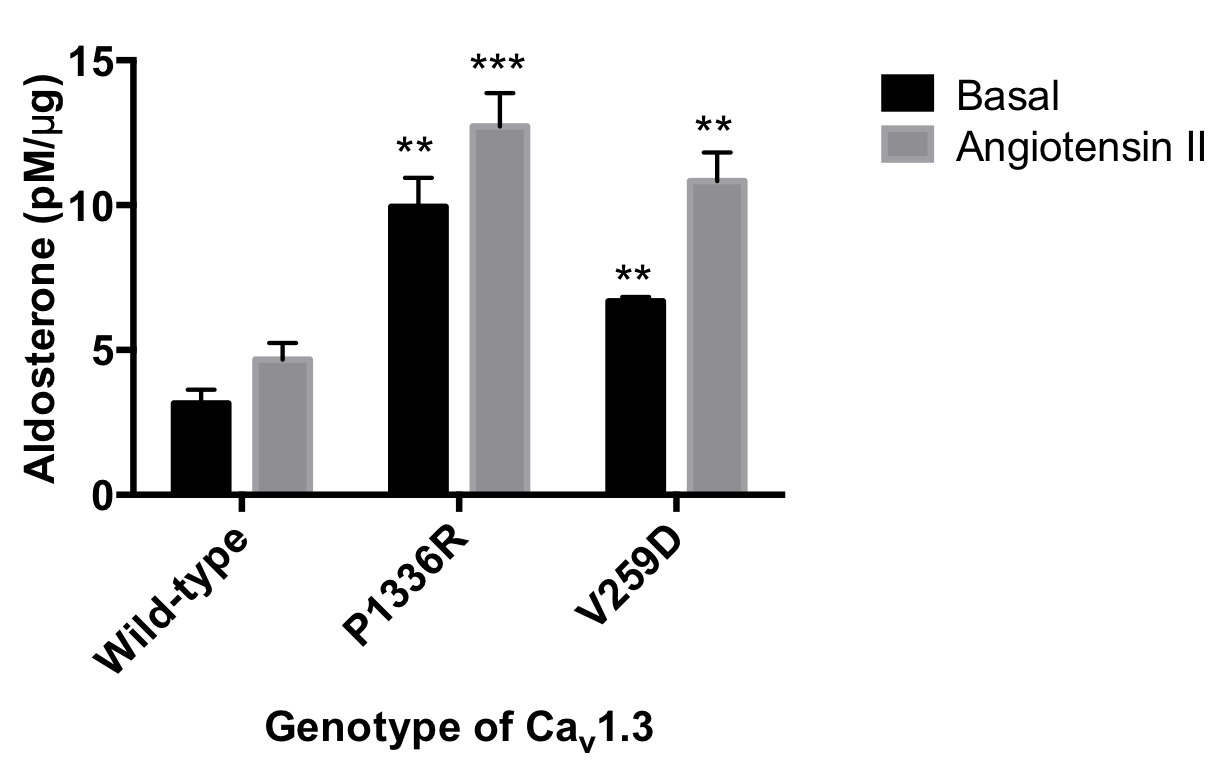


**Effect of angiotensin II on aldosterone secretion of H295R cells transfected with either wild-type (WT), P1336R, or V295D Ca_V_1.3 .**

Student *t*-test was used to calculate significance. ***P*<0.01 and ****P*<0.001, compared to wild-type.

Aldosterone results shown is an average (+SEM) of 3-4 biological replicates measured by the ELISA method.

**Supplementary Fig. 2: Effect of compound 8 and nifedipine on cortisol secretion in primary adrenal cells**


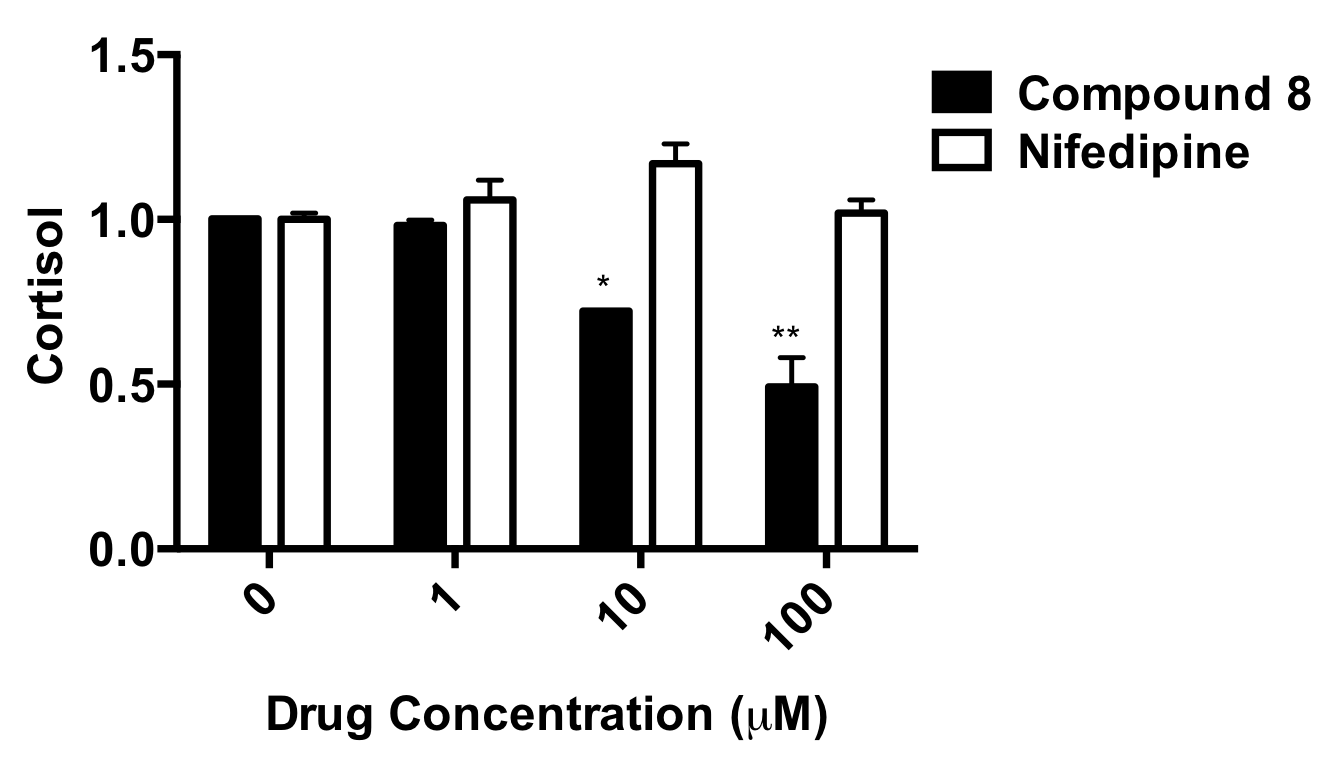


Compound **8** was investigated in adrenal cells adjacent to an aldosterone-producing adenoma (Patient ID: 181 and 184). Nifedipine was studied in a similar sample of adjacent adrenal cells (Patient ID: 182).

Student’s *t*-test was used to calculate statistical significance. **P*<0.05, ***P*<0.01, compared to baseline (0 M of treatment**)**. Each concentration was replicated 6 times within each individual patient samples. Cortisol results shown here are relative to basal level (0 M of treatment**)**.

**Supplementary Fig. 3: Localisation of wild-type Ca_v_1.3 and anti-Ca_v_1.3**


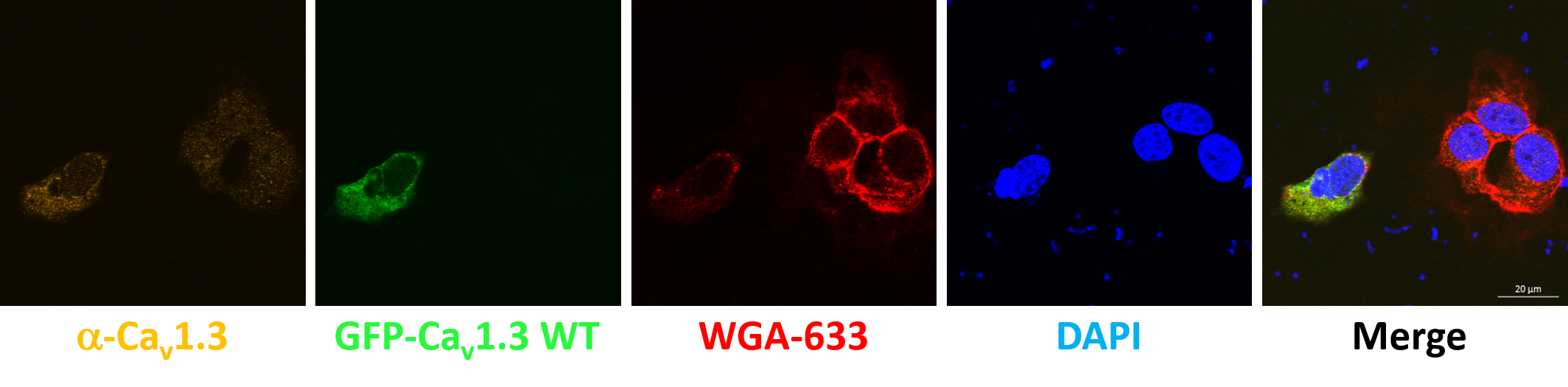


Immunofluorescence on H295R cells transfected with GFP-tagged Ca_v_1.3 WT. Endogenous and transfected Ca_v_1.3 was localized by immunofluorescent staining with anti-Ca_v_1.3 antibody (orange). GFP-Ca_v_1.3 WT (green), wheat-germ agglutinin (plasma membrane - red), and DAPI (nucleus-blue).

**Supplementary Fig. 4: Ca_V_1.3 expression in APA cells (low magnification).**

IHC of Ca_V_1.3 on FFPE adrenal sections were performed on three different types of APAs: (i-iii) ZG-like APAs without a Ca_V_1.3 mutation, (iv-vi) APAs with a Ca_V_1.3 mutation, and (vii-ix) APAs with a *KCNJ5* mutation. Immunostaining reveals a mixture of cytoplasm and membranous sublocalization in APA cells. This figure is a low magnification of Fig. 4.
